# Comparison of the efficacy of latanoprost versus dorzolamide/timolol fixed combination therapy in patients with pseudoexfoliative glaucoma according to glaucoma stage

**DOI:** 10.5935/0004-2749.2021-0230

**Published:** 2022-09-06

**Authors:** Pinar Sultan, Hulya Gungel, Furkan Ciftci

**Affiliations:** 1 Department of Ophthalmology, University of Health Sciences Istanbul Training and Research Hospital, Istanbul, Turkey; 2 Department of Ophthalmology, Sanliurfa Training and Research Hospital, Sanliurfa, Turkey

**Keywords:** Dorzolamide, Timolol, Glaucoma, Pseudoexfoliative glaucoma, Intraocular pressure, Latanoprost, Pharmaceutical preparations, Dorzolamide, Timolol, Glaucoma, Glaucoma pseudoesfoliativo, Pressão intraocular, Latanoprosta, Preparações farmacêuticas

## Abstract

**Purpose:**

Only a few trials have compared the intraocular pressure-lowering effects of prostaglandin analogs to carbonic anhydrase inhibitor plus beta-blocker fixed-dose combination therapy in patients with pseudoexfoliative glaucoma. Furthermore, the influence of the glaucoma stage on the intraocular pressure-lowering effects of these drug types has not been studied. The purpose of this study was to compare the IOP-lowering efficacy of latanoprost, a prostaglandin analog versus dorzolamide/timolol fixed combination, a carbonic anhydrase inhibitor plus beta-blocker fixed-dose combination therapy, in patients with pseudoexfoliative glaucoma based on glaucoma stage.

**Methods:**

The data of 32 eyes (32 patients) diagnosed with uniocular pseudoexfoliative glaucoma and treated with topical latanoprost (Group 1) or dorzolamide/timolol fixed combination (Group 2) were retrospectively assessed. The groups were subdivided into early and moderate-advanced stages. Patients’ demographics, baseline intraocular pressure, final intraocular pressure, and intraocular pressure difference (the difference between the baseline and final intraocular pressure) were determined from medical records and compared between groups and according to glaucoma stage.

**Results:**

The mean drug use duration was 17.7 ± 13.5 months. No significant differences in mean baseline intraocular pressure, mean final intraocular pressure and mean intraocular pressure difference between Groups 1 and 2. In Group 2, the mean intraocular pressure difference was significantly greater in patients with early versus moderate-advanced stage glaucoma (p=0.015). The difference, however, was not detected in Group 1. The mean intraocular pressure difference in early-stage glaucoma was significantly greater in Group 2 versus 1 (p=0.033).

**Conclusions:**

Latanoprost and dorzolamide/timolol fixed combination are effective treatments for newly diagnosed pseudoexfoliative glaucoma. In early-stage pseudoexfoliative glaucoma, greater intraocular pressure reduction was noted with dorzolamide/timolol fixed combination than with latanoprost; thus, dorzolamide/timolol fixed combination should be considered when a significant decrease in intraocular pressure is desired in early-stage glaucoma.

## Abstract (pt)

**Objetivo:**

Estudos limitados examinaram os efeitos de redução de pressão intraocular de análogos de prostaglandina versus inibidor de anidrase carbônica mais terapia de combinação de dose fixa beta-bloqueador em pacientes com glaucoma pseudoesfoliativo. Além disso, a influência do estágio de glaucoma nos efeitos de redução da pressão intraocular desses tipos de drogas não foi avaliada. Este estudo teve como objetivo comparar a eficácia de redução do IOP do latanoprosta, uma combinação fixa análoga de prostaglandina versus dorzolamida/timolol, um inibidor de anidrase carbônica mais terapia de combinação de dose fixa beta-bloqueador, em pacientes com glaucoma pseudoesfoliativo de acordo com o estágio de glaucoma.

**Métodos:**

Os dados de 32 olhos (32 pacientes) diagnosticados com glaucoma pseudoesfoliativo monocular e tratados com latanoprosta tópica (Grupo 1) ou combinação fixa de dorzolamida/timolol (Grupo 2) foram avaliados retrospectivamente. Os grupos foram subdivididos em estágios inicial e moderado-avançado. A demografia dos pacientes, a pressão intraocular da linha de base, a pressão intraocular final e a diferença de pressão intraocular (a diferença entre a pressão intraocular da linha de base e a pressão intraocular final) foram determinadas a partir dos prontuários médicos e comparadas entre os dois grupos e de acordo com o estágio de glaucoma.

**Resultados:**

A duração média do uso de drogas foi de 17,7 ± 13,5 meses. Nenhuma diferença significativa foi observada entre os grupos 1 e 2 para a média da pressão intraocularda linha de base, média da pressão intraocular final e média da diferença da pressão intraocular. No Grupo 2, a média da diferença da pressão intraocular foi significativamente maior em pacientes com glaucoma de estágio precoce versus moderado-avançado (p=0,015). No entanto, essa diferença não foi observada no Grupo 1. A média da diferença da pressão intraocular em glaucoma de estágio inicial foi significativamente maior no Grupo 2 versus 1 (p=0,033).

**Conclusões:**

Terapias com Latanoprosta e dorzolamida/timolol são tratamentos eficazes para glaucoma pseudoesfoliativo recém-diagnosticado. Observou-se em glaucoma pseudoesfoliativo de estágio inicial, uma maior redução da pressão intraocular com combinação fixa de dorzolamida/timolol do que com latanoprosta; assim, a combinação fixa de dorzolamida/timolol deve ser considerada quando uma diminuição significativa da pressão intraocular é almejada em glaucoma de estágio inicial.

## INTRODUCTION

Glaucoma, the leading cause of irreversible vision loss worldwide, is a progressive optic neuropathy characterized by retinal nerve fiber thinning and optic nerve head changes resulting in visual field defects^(1)^. Pseudoex foliative glaucoma (PXG) accounts for approximately 25% of all glaucoma types^(2)^, has a more severe clinical course, and is clinically and histopathologically distinct from primary open-angle glaucoma (POAG). The varied clinical course and fast progression of PXG necessitate more aggressive interventions^(3^, ^4^, ^5^, ^6)^ to control high intraocular pressure (IOP), the main risk factor for visual deterioration in glaucoma^(7)^. Lowering IOP with medication or surgery preserves visual function and decelerates the progression of glaucomatous visual field defects^(8^, ^9^, ^10)^.

Prostaglandin analogs (PGAs) promote aqueous humor outflow via the uveoscleral pathway and trabecular meshwork, while β-blockers and carbonic anhydrase inhibitors (CAI) slow aqueous humor production, thus reducing IOP^(11^, ^12^, ^13^, ^14)^. Inadequate IOP control with a single drug often warrants the addition of other anti-glaucomatous drugs^(15)^. In such cases, two-drug fixed-dose combinations in a single bottle are preferable over adding a second single drug for the ease of administration and minimization of preservative-related side effects. One of the well-known topical anti-glaucoma drugs is the fixed-dose carbonic anhydrase inhibitor plus beta-blocker (CAI+β blocker) combination, which is equally effective for IOP reduction as its components are used separately^(16)^.

When glaucoma is diagnosed, a patient might be in any stage of the disease. There is currently no consensus on a glaucoma classification based on severity. An increased C/D ratio suggests more severe glaucomatous changes^(17^, ^18^, ^19)^. Furthermore, in the staging of glaucoma, a variety of structural and functional testing methods are performed^(18^,^20)^. Many research groups have studied and compared the efficacy of CAI+β blocker fixed-dose combination therapy and PGAs inpatients with POAG^(21^, ^22^, ^23^, ^24)^. However, the IOP-lowering effects of these drugs may differ between PXG and POAG^(4)^, and only a few studies have compared PGA with CAI+β blocker fixed-dose combination therapy in PXG, none of which analyzed the influence of glaucoma stage on the IOP-lowering effect^(25^,^26)^.

The purpose of this study was to compare the efficacy of once-daily PGA (latanoprost) and twice daily CAI+β blocker fixed combination (dorzolamide/timolol) eye drops in patients with PXG, as well as to perform a sub-analysis of the IOP-lowering effects in early versus moderate-advanced stage PXG.

## METHODS

This retrospective analysis included the data of 32 eyes of 32 patients with a diagnosis of monocular PXG treated with either topical PGA (0.005% latanoprost) or topical CAI+β blocker fixed-dose combination therapy (2%/timolol 0.05% dorzolamide) who were followed up at the Glaucoma Unit of the University of Health Sciences Istanbul Training and Research Hospital, Department of Ophthalmology, between January 2014 and December 2019. The research design was approved by the Clinical Research Ethics Committee at the University of Health Sciences Istanbul Training and Research Hospital (Approval No. 1667) and the study protocol adhered to the tenets of the Declaration of Helsinki. The Ethics Committee waived the need for informed consent considering the retrospective nature of the study.

Every patient who refers to the Glaucoma Unit of the University of Health Sciences Istanbul Training and Research Hospital undergoes a complete ophthalmic examination after their detailed history is recorded. Best-corrected visual acuity was determined using the Snellen chart. Anterior segment examinations were performed using a biomicroscope. Vertical C/D ratios were determined using a 90-D lens. IOP measurements were performed by a trained physician with calibrated Goldmann applanation tonometers between 08:00 AM and 12:00 noon. Other data recorded included the central corneal thickness measured by using an ultrasonic pachymeter (UP-1000; Nidek, Japan); the visual field measurements were obtained using the Humphrey SITA 24/2 protocol (Humphrey Field Analyzer, Carl Zeiss Meditec AG, Jena, Germany) and anterior chamber angle examinations were performed by gonioscopy.

PXG was diagnosed based on the accumulation of pseudoexfoliative material at the pupillary border, angle, and/or anterior lens capsule, with high IOP measurements accompanied by glaucomatous optic neuropathy and visual field defects without treatment. The absence of pseudoexfoliation in the fellow eye was also confirmed by gonioscopy under miosis and biomicroscopy under wide mydriasis.

As in POAG treatment, PGA drops were preferred as the first line of treatment in PXG. The CAI+β blocker fixed-dose combination therapy was initiated in patients who did not want to use the PGA owing to the risk of cosmetic side effects such as iris color change, eyelash growth, and periocular skin pigmentation in one eye^(11)^. All patients used the same generic drug for both latanoprost and dorzolamide/timolol fixed combination.

The study inclusion criteria included the charts of patients aged >18 years with the diagnosis of monocular PXG and who had undergone treatment with topical latanoprost once in the evening or dorzolamide/timolol in fixed combination (DTFC) twice daily.

Patients with inflammatory eye diseases, retinal diseases, ocular infection in the last 3 months, history of glaucoma surgery or laser therapy, systemic diseases (heart or lung diseases), history of ocular trauma, and those using systemic β-blockers were excluded from the study. In addition, patients with large or small discs on optical coherence tomography images (Optovue OCT V 5.1, RTVue 100-2; Optovue, Fremont, CA, USA) were excluded. Patients with <3 months of follow-up and those lacking adequate archived information were also excluded. During the follow-up period, data collection from patients with drug exchange or in whom the second drug was added, was ended.

All 32 patients (32 eyes) with PXG were assigned to two groups based on the type of topical treatment. Group 1 included 12 patients who received latanoprost, and Group 2 included 20 patients who received DTFC. Both the groups were staged according to Hoddap-Parrish-Anderson criteria and divided into the early or moderate-advanced stage^(18)^.

The patient demographics, baseline IOP, final IOP, and IOP difference (the difference between the baseline IOP and final IOP) of the two groups were retrospectively analyzed. In addition, statistical sub-analysis, according to the glaucoma stage, was performed.

Statistical analyses were performed using the SPSS version 15.0 software (SPSS Inc., Chicago, IL, USA). The mean values and standard deviations of the investigated parameters were calculated. Student’s *t*-tests, Mann-Whitney *U-*tests, paired *t*-tests, and Wilcoxon Chi-squared tests were applied, as appropriate. Statistical significance was set at p<0.05.

When the retrospective power of the study was calculated based on pre-treatment intraocular pressure among the treatment groups (effect size 0.747), it was concluded to be 77%.

## RESULTS

A total of 21 (65.6%) of the 32 patients with PXG were in the early-stage, whereas 11 (34.4%) were in the moderate-advanced stage. There was no significant difference between the groups in terms of the glaucoma stage (p>0.05). The mean duration of drug use for all patients was 17.7 ± 13.5 months (range, 3-36 months).

Table 1 displays the demographic characteristics and ocular findings of the entire cohort and each study group. There were no significant differences in the mean duration of drug use or the affected eye between Groups 1 and 2 (all p>0.05). However, the mean age of Group 1 was significantly younger than that of Group 2 (p=0.011). The sex distributions were also significantly different between the groups, with Group 1 having more women than Group 2 (p=0.043). The number of patients who had previously undergone cataract surgery was significantly higher in Group 1 than in Group 2 (p=0.012), although there was no significant difference in the mean IOP across the groups based on cataract surgery (p>0.05).

**Table 1:** Demographic characteristics and ocular findings of the study groups

Characteristics	All patients	Group 1†	Group 2‡	p-value*
Age (years)	69.0 ± 7.1	65.0 ± 6.4	71.5 ± 6.5	**0.011**
Duration of Drug Use (months)	17.7 ± 13.5	16.8 ± 12.5	18.2 ± 14.3	0.770
Sex	14 (43.8)	8 (66.7)	6 (30.0)	**0.04**3
Female	18 (56.3)	4 (33.3)	14 (70.0)	
Male				
Eye	14 (43.8)	5 (41.7)	9 (45.0)	0.854
Right	18 (56.3)	7 (58.3)	11 (55.0)	
Left				
Cataract Surgery	23 (71.9)	12 (100)	11 (55.0)	**0.012**
Yes	9 (28.1)	0 (0)	9 (45.0)	
No				

Data are presented as the mean ± standard deviation or n (%);*

† patients with pseudoexfoliative glaucoma who were treated with a prostaglandin analog (latanoprost).

‡ patients with pseudoexfoliative glaucoma who were treated with a fixed-dose combination of carbonic anhydrase inhibitor+β blocker (dorzolamide-timolol).

Bold p-values: p<0.05.

**Figure 1:**
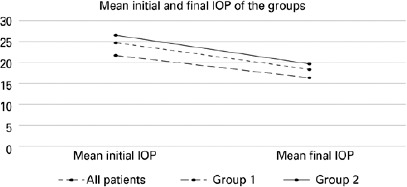
Mean baseline and final IOPs of the study groups. Group 1 included patients with pseudoexfoliative glaucoma who were treated with a prostaglandin analog (latanoprost). Group 2 included patients with pseudoexfoliative glaucoma who were treated with a carbonic anhydrase inhibitor+β blocker (dorzolamide-timolol) fixed-dose combination.

There were no statistically significant differences were identified in baseline IOP, final IOP, or IOP difference values between Groups 1 and 2 (p>0.05). The IOP decreased significantly after versus before treatment in both groups (both p<0.05) (Figure 1).

Table 2 displays the mean IOP differences according to sex and glaucoma stage. As for sex, there were no significant differences in the mean IOP difference between the sexes within each group or between groups based on sex (all p>0.05). Regarding the glaucoma stage, the mean IOP difference was significantly greater in Group 2 than in Group 1 for early-stage glaucoma (p=0.033). However, for moderate-advanced stage glaucoma, the mean IOP difference did not differ across groups (p>0.05). Although the mean IOP difference in Group 2 was significantly greater in patients with early versus moderate-advanced stage glaucoma (p=0.015), there was no significant difference in the mean IOP difference in Group 1 (p>0.05).

**Table 2:** Mean IOP differences according to the sex and stage of glaucoma

	Drug type				p-value*
	Group 1†		Group 2‡		
	Mean IOP difference (mmHg)¶		Mean IOP difference (mmHg)¶		
	Mean	SD	Mean	SD	
Female	5.63	2.97	6.33	6.15	0.844
Male	5.00	2.45	7.00	9.64	0.692
p-value**	0.663		0.649		
Early Stage	5.00	2.71	10.36	6.38	**0.033**
Moderate-advanced Stage	7.50	2.12	2.44	9.23	0.478
p-value**	0.226		**0.015**		

Data are presented as the mean ± standard deviation; *,**

† Consisted of patients with pseudoexfoliative glaucoma who were treated with a prostaglandin analog (latanoprost).

‡ Consisted of patients with pseudoexfoliative glaucoma who were treated with a fixed-dose combination of carbonic anhydrase inhibitor+β blocker (dorzolamide-timolol).

¶ IOP difference = final IOP - baseline IOP.

IOP= intraocular pressure; SD, standard deviation.

## DISCUSSION

Due to its poor prognosis, PXG is the most prevalent form of secondary open-angle glaucoma and requires greater control of IOP than POAG^(3^, ^4^, ^5^, ^6)^. In this study comparing the efficacy of once-daily latanoprost against twice daily DTFC in patients with newly diagnosed PXG, both drugs significantly reduced the mean IOP in patients with PXG by 17.7 ± 13.5 months.

The mean IOP was reduced considerably by 5.4 ± 2.7 mmHg (24.7%) in our patients treated with latano-prost for PXG at the final follow-up evaluation, and the quantitative IOP reduction did not differ by glaucoma stage. Previously, Parmaksiz et al.^(25)^ reported a mean IOP reduction of 9.3 ± 2.9 mmHg (36.5%) after 6 months of travoprost and 8.2 ± 1.2 mmHg (34%) after 6 months of latanoprost in PXG patients. After 2 months of lata-noprost treatment, Konstas et al.^(26)^ found that the mean IOP reduced from 31.2 ± 6.5 to 18.9 ± 4.1 mmHg (40.2% reduction).

At the final follow-up examination, the mean IOP in our PXG patients treated with DTFC was reduced by 6.8 ± 8.6 mmHg (25.5%). Similar studies on the efficacy of topical DTFC in PXG patients reported a mean IOP reduction of 45.2%^(25)^ and 42.8%, respectively^(26)^. Our IOP reduction percentages for both latanoprost and DTFC were lower than those reported in previous studies. This may be attributed to the longer follow-up period employed in the present study^(27)^. Another reason for this outcome might be the discontinuation of data collection from patients who had drug exchanges or had the second drug added. Despite the longer follow-up, the decrease of IOP in patients treated with latanoprost or DTFC was comparable.

Interestingly, we noticed that the IOP reduction was greater in patients treated with DTFC at the early versus moderate-advanced stage of PXG. This phenomenon did not occur in patients receiving latanoprost. This could be attributed to the fact that DTFC, through its aqueous-suppressant effect, reduces the outflow of the trabecular meshwork, and hence, cleaning of the fibrillar pseudo exfoliated material from the trabecular meshwork is reduced, and this mechanism might be impaired in the moderate-advanced stage of PXG^(27)^. After 2 months of treatment, Konstas et al.^(26)^ discovered no difference in mean IOP reduction between latanoprost and DTFC in newly diagnosed untreated PXG patients.

At 6 months following treatment initiation, DTFC reduced IOP more than PGA monotherapy (latanoprost, travoprost) in the study of PXG patients by Parmaksiz et al.^(25)^. However, Parmaksiz et al.^(25)^ excluded patients with a C/D ratio greater than 0.8, and no information regarding the patients’ glaucoma stages was provided. In this study, DTFC lowered the IOP more effectively than latanoprost in patients with early-stage PXG.

There are several limitations to our study that should be mentioned. First, while our research’s follow-up period was longer than those in the above-mentioned prospective studies, our study had a retrospective design and a small, heterogeneous sample size. Second, more patients receiving latanoprost than DTFC had previously undergone cataract surgeries. Cataract surgery might explain the observed adequate IOP control with PGA and the reduced need for fixed drug combinations. Prospective studies investigating the efficacy of the two types of drugs after phacoemulsification might offer further information on the effect of prior cataract surgery on IOP treated with PGA and CAI+β blocker fixed-dose combination therapy^(28^,^29)^.

In conclusion, both latanoprost and DTFC are effective treatments for newly diagnosed PXG. According to our findings, the IOP-lowering effects of DTFC are greater than are those of latanoprost in the early stages of PXG. As a result, in patients with early-stage PXG, DTFC should be preferred over PGA when greater IOP reduction is desired. Further prospective studies are needed to determine which anti-glaucomatous drug is more effective in patients with PXG according to the disease stage.
